# Error Analysis and Calibration Method of a Multiple Field-of-View Navigation System

**DOI:** 10.3390/s17030655

**Published:** 2017-03-22

**Authors:** Shuai Shi, Kaichun Zhao, Zheng You, Chenguang Ouyang, Yongkui Cao, Zhenzhou Wang

**Affiliations:** 1 State Key Laboratory of Precision Measurement Technology and Instruments, Tsinghua University, Beijing 100084, China; shi-s11@mails.tsinghua.edu.cn (S.S.); oycg13@mails.tsinghua.edu.cn (C.O.); caoyk13@mails.tsinghua.edu.cn (Y.C.); zz-wang13@mails.tsinghua.edu.cn (Z.W.); 2 Astronaut Center of China, Beijing 100094, China

**Keywords:** multiple field-of-view navigation system, imaging model, error analysis, checkerboard-fixed post-processing calibration

## Abstract

The Multiple Field-of-view Navigation System (MFNS) is a spacecraft subsystem built to realize the autonomous navigation of the Spacecraft Inside Tiangong Space Station. This paper introduces the basics of the MFNS, including its architecture, mathematical model and analysis, and numerical simulation of system errors. According to the performance requirement of the MFNS, the calibration of both intrinsic and extrinsic parameters of the system is assumed to be essential and pivotal. Hence, a novel method based on the geometrical constraints in object space, called checkerboard-fixed post-processing calibration (CPC), is proposed to solve the problem of simultaneously obtaining the intrinsic parameters of the cameras integrated in the MFNS and the transformation between the MFNS coordinate and the cameras’ coordinates. This method utilizes a two-axis turntable and a prior alignment of the coordinates is needed. Theoretical derivation and practical operation of the CPC method are introduced. The calibration experiment results of the MFNS indicate that the extrinsic parameter accuracy of the CPC reaches 0.1° for each Euler angle and 0.6 mm for each position vector component (1σ). A navigation experiment verifies the calibration result and the performance of the MFNS. The MFNS is found to work properly, and the accuracy of the position vector components and Euler angle reaches 1.82 mm and 0.17° (1σ) respectively. The basic mechanism of the MFNS may be utilized as a reference for the design and analysis of multiple-camera systems. Moreover, the calibration method proposed has practical value for its convenience for use and potential for integration into a toolkit.

## 1. Introduction

**Figure 1:**
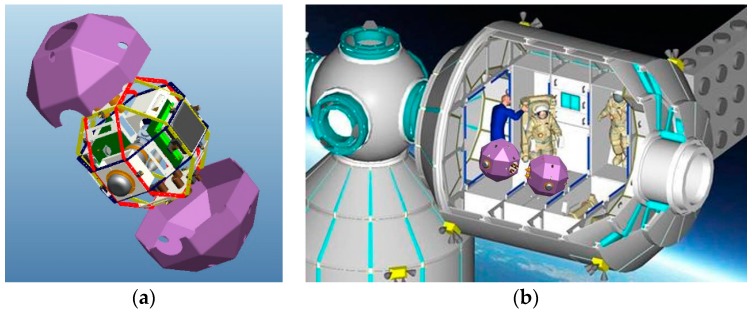
(**a**) 3D model and (**b**) flying sketch of the SITSS.

**Figure 2:**
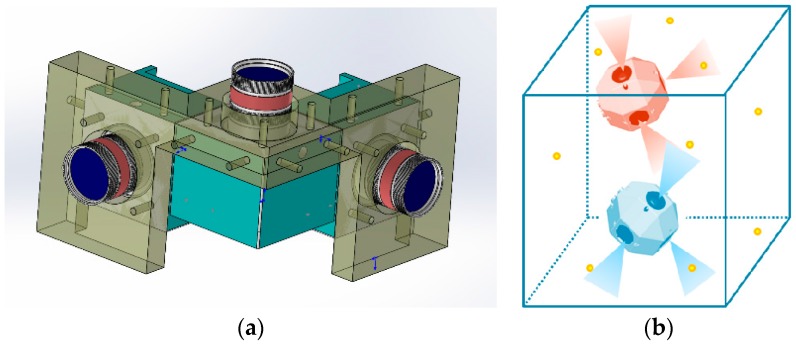
(**a**) Prototype and (**b**) navigation sketch of the MFNS.

According to its space program schedule, China will launch the Tiangong Space Station in the next few years. To make use of the microgravity environment and conduct flight experiments and on-orbit service inside the space station, the Spacecraft Inside Tiangong Space Station (SITSS) is proposed (Figure 1). The SITSS aims to offer a platform for on-orbit-distributed satellite flight tests and serve as free-flying robots for environmental monitoring and astronaut assistance. Hence, an accurate, fast, and fully autonomous navigation system is expected. Nowadays, computer vision is widely used in the observation [1], measurement [2], entertainment [3], and navigation [4] fields because of the rich information it acquired from the surrounding environment, high precision, and potential for intelligence. To achieve the intended function for SITSS, a multiple field-of-view navigation system (MFNS) for SITSS (Figure 2) is built via computer vision.

Parameter calibration is the fundamental problem in computer vision and has been the focus of research since it was first proposed. An earlier work can be found dating back to the 1970s [5], and a large set of works are dedicated to camera calibration methods and calibration model analyses. Calibration methods that require beacon coordinates in the 3D world can be found in [6,7,8]. Other automatic methods were proposed by Tsai [9], Strum [10], and Heikkila [11] et al., all of whom utilized known objects as calibration references. Zhang’s method [12] published in 2000 observed an easy and accurate calibration method based on a planar checkerboard placed in different locations in the field-of-view (FOV) of a camera. However, those automatic methods are inconvenient to use in large FOV applications when the size of the reference object is limited. Aside from those methods, some approaches focus on calibration using vanishing points based on geometric relationships [13,14,15]. A large amount of literature on calibrating single cameras or vision systems based on extension, optimization, and adaption of methods applicable to different situations have been introduced as well [16,17,18]. Those methods are also applicable in other fields, for instance, the error analysis and calibration method of star trackers [19].

The effective and accurate intrinsic and extrinsic parameters of conventional single cameras can be recognized in many ways. The position and attitude relationship between cameras is a concern for binocular or multiple camera vision systems, such a relationship can be obtained based on homography because the cameras share a substantial overlap [20,21]. However, obtaining this relationship becomes a challenge when the cameras of a system do not share a visual field. In recent years, researchers have come up with several remarkable ideas for non-overlapping camera systems, some of them use mirrors and a calibration target [22,23], some use rigid motion constraints [24,25] while some use a combination of those two methods [26,27]. Unfortunately, these methods seem incapable of solving the problem of MFNS calibration directly, as the attitudes and positions of the cameras with respect to the MFNS coordinate are indispensable while difficult to obtain through either existing camera calibration methods or mechanical measurements.

In the present paper, we attempt to solve this problem by introducing an imaging model of the MFNS and its error analysis. A novel calibration method is proposed on this imaging model, which meets the requirement of MFNS and other similar systems. Our experiment indicates that the MFNS can reach an expected calibration accuracy by utilizing the method proposed. We introduce the mathematical model of the MFNS in Section 2. The error analysis of the system and the simulation result analysis are discussed in Section 3. The checkerboard-fixed post-processing calibration (CPC) method for MFNS parameters is proposed in Section 4, and a verification navigation experiment is conducted in Section 5. Finally, the conclusions are presented in Section 6.

## 2. Mathematical Model

### 2.1. Imaging Model

**Figure 3:**
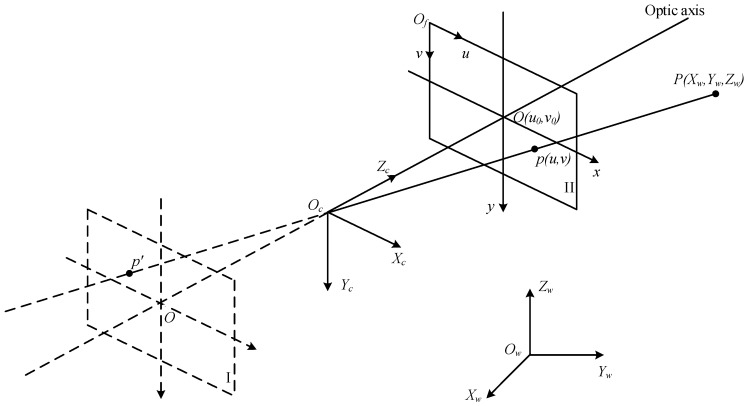
Pinhole imaging model.

The pinhole model is commonly used for ideal mapping of the 3D points of the world to the 2D image generated by a camera [6,28]. Figure 3 displays the perspective projection performed by a pinhole camera. The world point *P* is projected through the projection center of the lens to the point *p* in the image plane I. Plane II is the symmetry plane of I, which is introduced to facilitate the analysis. The relationship between *P* (*X_w_*, *Y_w_*, *Z_w_*) and its image *p* (*u*, *v*) is as follows:

(1) $$\begin{array}{l} Z_{c}\left[\begin{array}{c} u\\ v\\ 1 \end{array}\right]=\left[\begin{array}{ccc} 1/dx & s & u_{0}\\ 0 & 1/dy & v_{0}\\ 0 & 0 & 1 \end{array}\right]\left[\begin{array}{cccc} f & 0 & 0 & 0\\ 0 & f & 0 & 0\\ 0 & 0 & 1 & 0 \end{array}\right]\left[\begin{array}{cc} R & T\\ 0^{T} & 1 \end{array}\right]\left[\begin{array}{c} X_{W}\\ Y_{W}\\ Z_{W}\\ 1 \end{array}\right]=\left[\begin{array}{cccc} \alpha_{x} & \gamma & u_{0} & 0\\ 0 & \alpha_{y} & v_{0} & 0\\ 0 & 0 & 1 & 0 \end{array}\right]\left[\begin{array}{cc} R & T\\ 0^{T} & 1 \end{array}\right]\left[\begin{array}{c} X_{W}\\ Y_{W}\\ Z_{W}\\ 1 \end{array}\right]\\ =M_{1}M_{2}X_{W}=MX_{W} \end{array}$$

where:

*Z_c_* is the optic axis coordinate of point *P*,
*dx* is the ratio coefficient in the *x* direction,
*dy* is the ratio coefficient in the *y* direction,
*s* and *γ* are the non-orthogonal factor of axes of the image coordinate,
(*u*_0_*, v*_0_) is the pixel coordinate of the camera principal point,
*f* is the principal distance of the camera,
***R*** is the 3 × 3 rotation matrix,
***T*** is the 3D translation vector,
*α_x_ = f/dx* and *α_y_ = f/dy* are the respective scale factors of the *u*-axis and *v*-axis of the image coordinate,
***M*_1_** and ***M*_2_** are the intrinsic parameter matrix and extrinsic parameter matrix respectively, and
***M = M*_1_**·***M*_2_** is the perspective projection transform matrix.

In the actual imaging system, the displacement of the principal point, focal length deviation, distortion, and other error factors should be considered on the basis of ideal pinhole imaging model (see Section 4.1).

### 2.2. MFNS Model

Three industrial cameras whose optic axes are approximately perpendicular to one another are the main components of the MFNS (Figure 2). This arrangement is adopted for immediate and accurate navigation performance of the SITSS, because it presents six degrees of freedom of free flight inside the space station. The three cameras form a generalized monocular vision system while the FOV is extended, and the accuracy in three directions is optimized compared with traditional monocular vision systems. Based on the imaging model of a single camera (Equation (1)), the imaging model of the MFNS is given as follows:

(2) $$Z_{ck}\left[\begin{array}{c} u_{k}\\ v_{k}\\ 1 \end{array}\right]=\left[\begin{array}{cccc} \alpha_{xk} & \gamma_{k} & u_{0k} & 0\\ 0 & \alpha_{yk} & v_{0k} & 0\\ 0 & 0 & 1 & 0 \end{array}\right]\left[\begin{array}{cc} C_{b}^{k} & 0\\ 0^{T} & 1 \end{array}\right]\left[\begin{array}{cc} C_{w}^{b} & r_{bw}^{b}-r_{bk}^{b}\\ 0^{T} & 1 \end{array}\right]\left[\begin{array}{c} X_{Wi}\\ Y_{Wi}\\ Z_{Wi}\\ 1 \end{array}\right]$$

where *k* = *x*, *y*, *z* represents camera X, Y, and Z, respectively; *i* = *1*, *2*, *3*,…represents the number of beacons observed, $C_{b}^{k}$ is the rotation matrix form of the MFNS *body* coordinate to the camera *k* coordinate, $C_{w}^{b}$ is the rotation matrix from the *world* coordinate to the MFNS *body* coordinate, and $r_{bw}^{b}={\left(t_{x},t_{y},t_{z}\right)}^{T}$ and $r_{bk}^{b}={\left(r_{xk},r_{yk},r_{zk}\right)}^{T}$ are the translation vectors from the origin of the MFNS *body* coordinate to the origins of the *world* coordinate and camera *k* coordinates respectively, which are expressed in the MFNS *body* coordinate.

Although a common way to model a set of rigidly attached cameras is using a generalized camera model, which find its way in many applications such as motion estimation, image reconfiguration, etc. [29,30,31,32], equation.4 is a practical model of the MFNS, which is an extension of the commonly used pinhole model. Coordinate transformation is simply applied during the derivation, which makes it simple and reliable. Moreover, the model of Equation (2) is convenient for navigation applications in our future work, as the rotation matrices and translation vectors have clear physical meanings, which are exactly the 6-DOF navigation parameters (three-axis attitude and position). Thus measurement equations can be built directly based on Equation (2). Let $A^{k}=\left[\begin{array}{cccc} \alpha_{xk} & \gamma_{k} & u_{0k} & 0\\ 0 & \alpha_{yk} & v_{0k} & 0\\ 0 & 0 & 1 & 0 \end{array}\right]\left[\begin{array}{cc} C_{b}^{k} & 0\\ 0^{T} & 1 \end{array}\right]={\left(a_{ij}^{k}\right)}_{3\times 4}$ and $C_{w}^{b}={\left(r_{ij}\right)}_{3\times 3}$. We therefore get:

(3) $$\left\{ \begin{array}{l} \left(a_{11}^{k}-a_{31}^{k}u_{k})(r_{11}X_{Wi}+r_{12}Y_{Wi}+r_{13}Z_{Wi}+t_{x}-r_{xk})+(a_{12}^{k}-a_{32}^{k}u_{k})(r_{21}X_{Wi}+r_{22}Y_{Wi}+r_{23}Z_{Wi}+t_{y}-r_{yk}\right)\\ +(a_{13}^{k}-a_{33}^{k}u_{k})(r_{31}X_{Wi}+r_{32}Y_{Wi}+r_{33}Z_{Wi}+t_{z}-r_{zk})=0\\ \left(a_{21}^{k}-a_{31}^{k}v_{k})(r_{11}X_{Wi}+r_{12}Y_{Wi}+r_{13}Z_{Wi}+t_{x}-r_{xk})+(a_{22}^{k}-a_{32}^{k}v_{k})(r_{21}X_{Wi}+r_{22}Y_{Wi}+r_{23}Z_{Wi}+t_{y}-r_{yk}\right)\\ +(a_{23}^{k}-a_{33}^{k}v_{k})(r_{31}X_{Wi}+r_{32}Y_{Wi}+r_{33}Z_{Wi}+t_{z}-r_{zk})=0 \end{array}\right.$$

Equation (5) is the practical imaging model of the MFNS. The pose of the MFNS (six unknowns) is possibly obtained by solving a Perspective-N-Point problem [33,34] when plenty of beacons are observed.

## 3. Error Analysis

The MFNS measurements are the coordinates of the images of navigation reference beacons. In the imaging model (Equation (2)), the error in beacon positions, intrinsic parameter calibration error of cameras, pose (with respect to the MFNS) calibration error of cameras, and algorithm error are the factors affecting the measurement accuracy of the system. The main focus of this research is the influence of the system parameter error on the measurement error. The algorithm error will be studied in future work.

To facilitate the equation derivation and error analysis, the following discussion is based on the assumption that $C_{b}^{z}=I_{3}$ and $r_{bz}^{b}=0$. Given that cameras X, Y, and Z are equivalent, only the measurement error of camera Z is calculated and simulated as follows:

(1) Beacon position error

The pixel coordinate (*u*, *v*) of a beacon observed by camera Z is given by:

(4) $$\begin{array}{l} u=\alpha_{x}\frac{r_{11}X_{W}+r_{12}Y_{W}+r_{13}Z_{W}+t_{x}}{r_{31}X_{W}+r_{32}Y_{W}+r_{33}Z_{W}+t_{z}}+u_{0}=\alpha_{x}\frac{X_{c}}{Z_{c}}+u_{0}\\ v=\alpha_{y}\frac{r_{21}X_{W}+r_{22}Y_{W}+r_{23}Z_{W}+t_{y}}{r_{31}X_{W}+r_{32}Y_{W}+r_{33}Z_{W}+t_{z}}+v_{0}=\alpha_{y}\frac{Y_{c}}{Z_{c}}+v_{0} \end{array}$$

where (*X_c_*, *Y_c_*, *Z_c_*) is the coordinate of that beacon in the camera Z coordinate.

Suppose the position of a beacon is inaccurate and its coordinate is (*X_w_* + Δ*X_w_*, *Y_w_* + Δ*Y_w_*, *Z_w_* + Δ*Z_w_*), then the measurement of the pixel coordinate is given by:

(5) $$\begin{array}{l} u_{beacon}^{\prime }=\alpha_{x}\frac{r_{11}(X_{W}+\Delta X_{W})+r_{12}(Y_{W}+\Delta Y_{W})+r_{13}(Z_{W}+\Delta Z_{W})+t_{x}}{r_{31}(X_{W}+\Delta X_{W})+r_{32}(Y_{W}+\Delta Y_{W})+r_{33}(Z_{W}+\Delta Z_{W})+t_{z}}+u_{0}=\alpha_{x}\frac{X_{cb}^{\prime }}{Z_{cb}^{\prime }}+u_{0}\\ v_{beacon}^{\prime }=\alpha_{y}\frac{r_{11}(X_{W}+\Delta X_{W})+r_{12}(Y_{W}+\Delta Y_{W})+r_{13}(Z_{W}+\Delta Z_{W})+t_{y}}{r_{31}(X_{W}+\Delta X_{W})+r_{32}(Y_{W}+\Delta Y_{W})+r_{33}(Z_{W}+\Delta Z_{W})+t_{z}}+v_{0}=\alpha_{y}\frac{Y_{cb}^{\prime }}{Z_{cb}^{\prime }}+v_{0} \end{array}$$

Thus, the error of measurement is as follows:

(6) $$\begin{array}{l} \Delta u_{b}=u_{beacon}^{\prime }-u=\alpha_{x}(\frac{X_{cb}^{\prime }}{Z_{cb}^{\prime }}-\frac{X_{c}}{Z_{c}})\\ \Delta v_{b}=v_{beacon}^{\prime }-v=\alpha_{y}(\frac{Y_{cb}^{\prime }}{Z_{cb}^{\prime }}-\frac{Y_{c}}{Z_{c}}) \end{array}$$

Let ***r***_1_ = (*r*_11_, *r*_12_, *r*_13_)^T^, ***r***_2_ = (*r*_21_, *r*_22_, *r*_23_)^T^, ***r***_3_ = (*r*_31_, *r*_32_, *r*_33_)^T^, and Δ***P****_w_* = (Δ*X_w_*, Δ*Y_w_*, Δ*Z_w_*)^T^. Equation (6) could be rewritten as:

(7) $$\begin{array}{l} \Delta u_{b}=\alpha_{x}(\frac{X_{c}+r_{1}\cdot \Delta P_{w}}{Z_{c}+r_{3}\cdot \Delta P_{w}}-\frac{X_{c}}{Z_{c}})\\ \Delta v_{b}=\alpha_{y}(\frac{Y_{c}+r_{2}\cdot \Delta P_{w}}{Z_{c}+r_{3}\cdot \Delta P_{w}}-\frac{Y_{c}}{Z_{c}}) \end{array}$$

The Monte Carlo (MC) method is used to study the relationship between the beacon position error and the measurement error. The parameters of the method are shown in Table 1. Only the pixel error of *u* is simulated because the properties of the pixels in the two directions (*u*, *v*) [or (*X_c_*, *Y_c_*)] are the same. The MFNS coordinate is taken as the reference frame because of the imaging progress. Specifically, the MFNS is fixed at the origin of the world coordinate (see the extrinsic parameter of the MFNS in Table 1), and the beacons are in any possible position (see beacon position in Table 1). As a result, the simulation model is simplified and the result is more convenient for observation.

**Figure 4:**
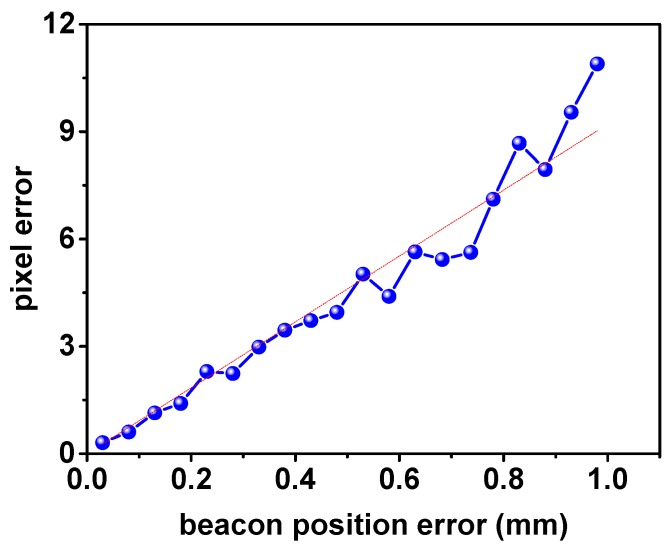
MC simulation result of the pixel error caused by the beacon error.

Figure 4 shows the simulation result, the dots in the figure stand for the root-mean-square error of the MC results, the same below. The pixel error increases linearly with the approximate beacon position error.

(2) Camera intrinsic parameter error

On the basis of the pinhole imaging model (Equation (2)), the influence of the principal point displacement, the focal length error, and the lens distortion should be considered in the practical vision measurement system. These parameters are the intrinsic parameters of a camera, whose calibration is one of the key problems in vision research. At present, a reasonable choice of calibration method ensures that the imaging accuracy reaches the sub-pixel level.

(3) Relative position error between camera and MFNS

When multiple cameras are integrated in the MFNS, a relative position error between each camera coordinate and the system body coordinate can occur, known as the error of the translation vector ***T*** in the imaging model. Let ***T′***= (*t_x_ +* Δ*t_x_, t_y_* + Δ*t_y_, t_z_* + Δ*t_z_*)^T^; it is obtained according to the imaging model:

(8) $$\begin{array}{l} \Delta u_{p}=u_{position}^{\prime }-u=\alpha_{x}(\frac{X_{c}+\Delta t_{x}}{Z_{c}+\Delta t_{z}}-\frac{X_{c}}{Z_{c}})\\ \Delta v_{p}=v_{position}^{\prime }-v=\alpha_{y}(\frac{Y_{c}+\Delta t_{y}}{Z_{c}+\Delta t_{z}}-\frac{Y_{c}}{Z_{c}}) \end{array}$$

**Figure 5:**
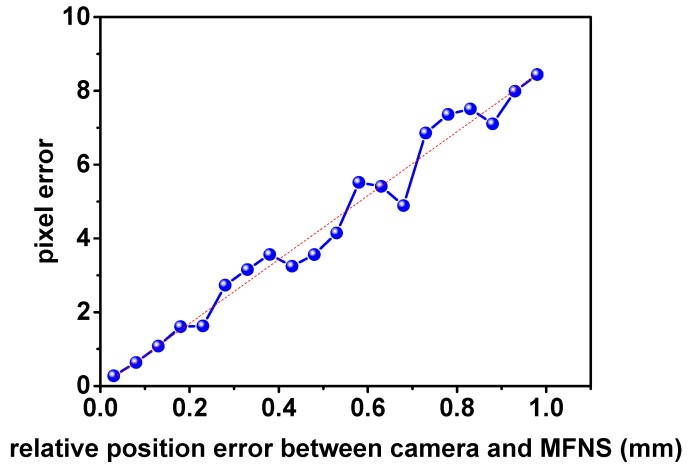
MC simulation result of pixel error caused by the relative position error.

The result of the MC simulation of the pixel error is shown in Figure 5, while the parameters are set as shown in Table 1.

(4) Relative attitude error between cameras and MFNS

Similar to the relative position error, an error inevitably occurs in the rotation matrix between the camera coordinate system and the MFNS coordinate, known as the error of attitude of Euler angle *φ*, *θ,* and *ψ* in the imaging model. Let ***Θ′*** = (*φ +* Δ*φ,* *θ* + Δ*θ,* *ψ* + Δ*ψ*)^T^. The rotation matrix in the imaging model (Euler angles are defined as 2-1-3 rotation in this paper) can be rewritten as follows:

(9) $$\begin{array}{l} r_{11}^{\prime }=\cos(\psi +\Delta \psi )\cos(\theta +\Delta \theta )+\sin(\psi +\Delta \psi )\sin(\theta +\Delta \theta )\sin(\varphi +\Delta \varphi )\\ r_{12}^{\prime }=\sin(\psi +\Delta \psi )\cos(\varphi +\Delta \varphi )\\ r_{13}^{\prime }=-\cos(\psi +\Delta \psi )\sin(\theta +\Delta \theta )+\sin(\psi +\Delta \psi )\cos(\theta +\Delta \theta )\sin(\varphi +\Delta \varphi )\\ r_{21}^{\prime }=-\sin(\psi +\Delta \psi )\cos(\theta +\Delta \theta )+\cos(\psi +\Delta \psi )\sin(\theta +\Delta \theta )\sin(\varphi +\Delta \varphi )\\ r_{22}^{\prime }=\cos(\psi +\Delta \psi )\cos(\varphi +\Delta \varphi )\\ r_{23}^{\prime }=\sin(\psi +\Delta \psi )\sin(\theta +\Delta \theta )+\cos(\psi +\Delta \psi )\cos(\theta +\Delta \theta )\sin(\varphi +\Delta \varphi )\\ r_{31}^{\prime }=\cos\varphi \sin(\theta +\Delta \theta )\\ r_{32}^{\prime }=-\sin(\varphi +\Delta \varphi )\\ r_{33}^{\prime }=\cos(\theta +\Delta \theta )\cos(\varphi +\Delta \varphi ) \end{array}$$

Let ***r′***_1_ = (*r′*_11_, *r*′_12_, *r′*_13_)^T^, ***r′***_2_ = (*r′*_21_, *r′*_22_, *r′*_23_)^T^, ***r′***_3_ = (*r′*_31_, *r′*_32_, *r′*_33_)^T^, ***P****_w_* = (*X_w_*, *Y_w_*, *Z_w_*)^T^. Thus, we get

(10) $$\begin{array}{l} \Delta u_{a}=u_{attitude}^{\prime }-u=\alpha_{x}(\frac{r_{1}^{\prime }\cdot P_{w}}{r_{3}^{\prime }\cdot P_{w}}-\frac{X_{c}}{Z_{c}})\\ \Delta v_{a}=v_{attitude}^{\prime }-v=\alpha_{y}(\frac{r_{2}^{\prime }\cdot P_{w}}{r_{3}^{\prime }\cdot P_{w}}-\frac{Y_{c}}{Z_{c}}) \end{array}$$

**Figure 6:**
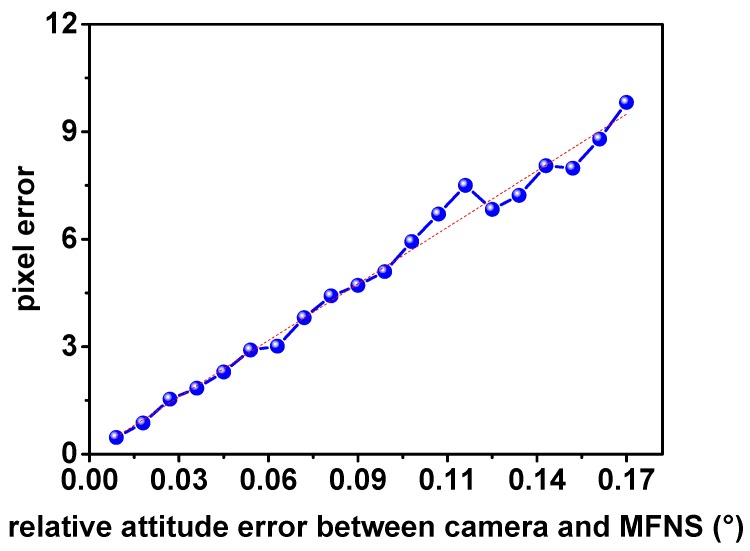
MC simulation result of pixel error caused by the relative attitude error.

The result of the MC simulation of pixel error is shown in Figure 6, while the parameters are set as shown in Table 1.

In Figure 4, Figure 5 and Figure 6, the pixel error grows approximately linearly with the beacon error and the MFNS extrinsic parameter error (including the relative position error and attitude error between cameras and the MFNS), such outcome is regarded as a system error. System errors have a serious influence on MFNS performance compared to the intrinsic parameter calibration errors of cameras. The simulation results show that the relative position and attitude of the system must be calibrated to an accuracy of <1 mm and <0.17° respectively if the observation error of the MFNS is expected to be at several pixels. This work attempts to find a solution for this challenging problem.

## 4. System Calibration Based on Geometrical Constraints in Object Space

### 4.1. CPC Method for MFNS

To achieve the accuracy requirements of the MFNS, the system parameters need to be calibrated, including the intrinsic and extrinsic parameters of each camera, namely, the transformation between the camera coordinates and the MFNS coordinate system. Accurate estimation of the camera parameters is the prerequisite for identification, measurement, and navigation in most computer vision applications. Therefore, scholars have carried out in-depth, extensive research on the calibration of camera intrinsic parameters to solve the related theoretical problems and put forward a variety of mature methods. Both intrinsic and extrinsic parameters must be accurately calibrated according to the error analysis of the MFNS in Section 3. However, at present, most methods mainly deal with the calibration of a single camera. For binocular vision systems, the common method is calibrating first the intrinsic parameters of each camera and then calculating the transformation between the two camera coordinates on the basis of the correspondence of images of the shared overlap.

**Figure 7:**
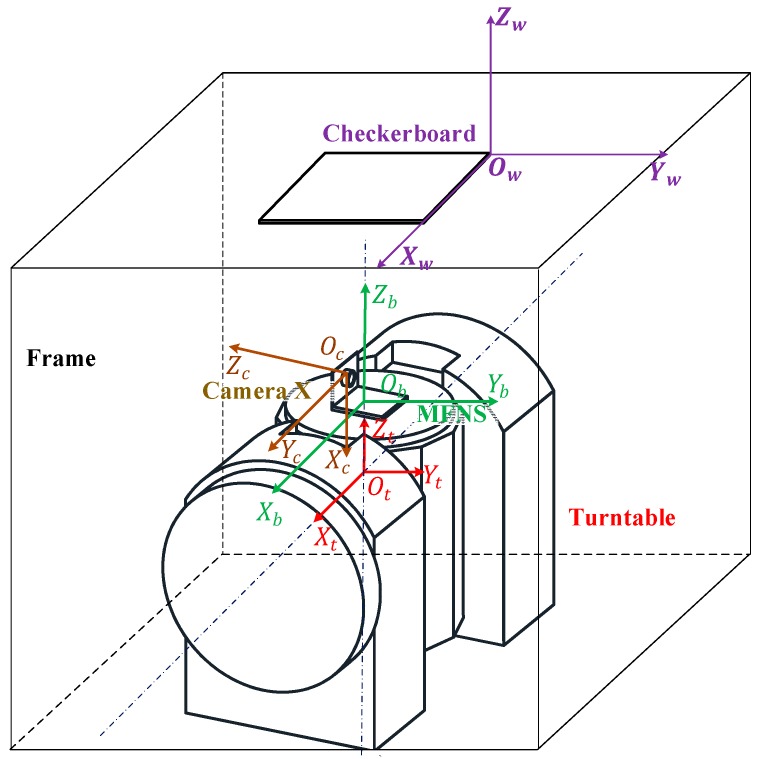
Calibration architecture of MFNS.

However, the FOV of the cameras is discrete for MFNS calibration; thus, the cameras cannot observe a certain object simultaneously. Therefore, the existing single camera/binocular system calibration method cannot obtain the transformation between the camera coordinates. It is notable that there are a number of studies on calibration of non-overlapping cameras, these studies give good solutions for calibration of common non-overlapping cameras, but most of them are unable to satisfy the requirement of the MFNS calibration. The reason is that the installation matrix between the cameras and the MFNS likewise needs to be calibrated to achieve precise navigation and that is the prerequisite for the application of the MFNS on a carrier, but this problem tends to be ignored or less considered in computer vision application. To solve this problem, the geometrical constraints in the object space are utilized to build a transformation relationship between the FOV-separated cameras and the MFNS. The checkerboard is fixed and photographs are taken by each camera. Then, a high-accuracy turntable is utilized to control the pose of the MFNS and provide a dependable reference during the calibration process. All the coordinates involved in the calibration method are right-handed Cartesian coordinates (Figure 7) as follows:

1. MFNS body coordinate *O_b_X_b_Y_b_Z_b_*: fixed on the system frame and defined for ease of use.
2. Turntable coordinate *O_t_X_t_Y_t_Z_t_*: *O_t_* is the center of the turntable, *Z_t_* points to the forward of the turntable main axis, and *X_t_* points to the forward of the turntable auxiliary axis.
3. World coordinate *O_w_X_w_Y_w_Z_w_*: defined by the checkerboard according to the single camera calibration method, where *O_w_* is the corner of the checkerboard, and *X_w_*, and *Y_w_* are parallel to the edge of the checkerboard grid.
4. Coordinates of cameras X, Y, and Z: defined based on the imaging model in Section 2.1 and recorded respectively as *O_x_X_x_Y_x_Z_x_*, *O_y_X_y_Y_y_Z_y_*, and *O_z_X_z_Y_z_Z_z_*.

The superscripts and subscripts *b*, *t*, *w*, *x*, *y,* and *z* represent the coordinate systems of the MFNS, turntable, world, and cameras X, Y, and Z, respectively.

All physical quantities that should be calibrated are defined as follows:

1. Intrinsic parameter matrix ***A****_k_* and distortion coefficient *kc*_1*k*_–*kc*_5*k*_;
2. Rotation matrix $C_{k}^{b}$ between camera *k* coordinate and the MFNS; and
3. Vector ***r****_i_* from camera principal points *O_cx_*, *O_cy_*, and *O_cz_* to the origin of the MFNS coordinate system *O_b_*_,_ where *k* = *x*, *y,* and *z* stand for cameras X, Y, and Z, respectively.

Note that the intrinsic and extrinsic parameters of cameras X, Y, and Z can be estimated using the single camera calibration method, for instance the widely used Zhang’s method [12]. That is, the relationship between the camera systems and the world coordinate system is obtained after Zhang’s calibration. When the relationship between the MFNS coordinate system and the world system is established, the maximum likelihood estimation of the extrinsic parameters through the single camera calibration method can be utilized to carry out the solution for $C_{k}^{b}$ and ***r****_i_*. Based on the ideas above, *checkerboard-fixed post-processing calibration method* is proposed, which can achieve MFNS calibration combined with most single camera calibration methods. In this paper, Zhang’s method is utilized for single camera calibration. The theoretical derivation and operation details are seen in Appendix A. Note that the MFNS coordinate is defined as follows considering ease of use: the origin of the coordinate is the center of the MFNS bottom surface, and the the *X_b_* axis and *Z_b_* axis of the MFNS coordinate are parallel to the respective *X_t_* axis and *Z_t_* axis of the turntable coordinate when the MFNS is mounted to the turntable. This coordinate definition is ensured by operations of finish, drilling threaded holes and locating holes performed on the bottom surface of the MFNS.

**Figure 8:**
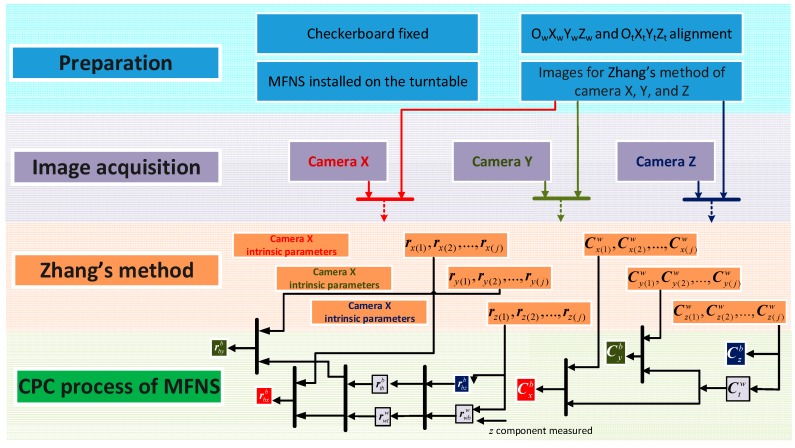
Process of MFNS calibration method.

Figure 8 shows the process of our calibration method. The *preparation* needs to be done before system calibration:

1. The checkerboard should be fixed in an appropriate location that can be observed by the cameras while the MFNS rotates with the turntable.
2. Several pictures of the checkerboards that meet the requirements of the Zhang’s method are taken by cameras X, Y, and Z.
3. Alignment of the coordinates should be done subsequently so that the rotation matrix from the turntable coordinate to the world coordinate is approximate to an identity matrix.

Afterwards, the turntable is controlled manually to rotate around both *X_t_* and *Z_t_* axes for checkerboard *image acquisition* by each camera, and the attitude of the turntable corresponding to each image is recorded. With the images prepared earlier and obtained, the maximum likelihood solutions of each camera’s parameters are given by *Zhang’s calibration method* (note that the previously prepared images are necessary to obtain good results using Zhang’s method, because the pictures of the checkerboard taken by the MFNS during rotation may not satisfy the diversity of the different checkerboard poses for Zhang’s method.) Finally, all the parameters needed are evaluated based on Zhang’s method results and the *CPC process of the MFNS* introduced. The contrast between the proposed method and the hand-eye calibration is as follows: In hand-eye calibration, the robot arm and cameras are attached, as the motion of the robot arm (hand) is known, the transformation between the cameras’(eye) coordinates and the base coordinate can be calculated through multiple poses and images taken corresponding to those poses. In our work, the “eye” is the MFNS and “hand” is the turntable, while the “eye” will be removed from the “hand” after calibration and used in other situations.

### 4.2. Calibration Results

**Figure 9:**
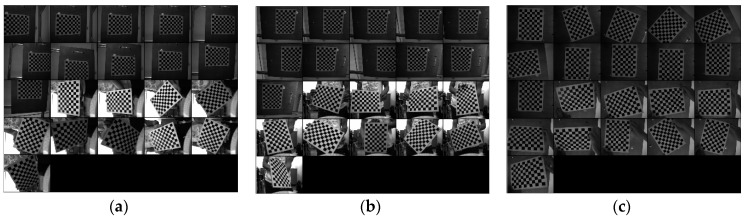
Images for calibration of camera X (**a**), camera Y (**b**), and camera Z (**c**) using Zhang’s method.

An MFNS calibration is conducted using the method proposed. We use the 902E-1 two-axis testing turntable produced by Beijing Precision Engineering Institute for Aircraft Industry (Beijing, China). The turntable is aligned before MFNS calibration, and the accuracy of its angular position is 8″ in the direction of both axes. The cameras integrated in the MFNS are Daheng Image DH-HV1310FM (resolution 1280 × 1024, 18.6 frame/s under highest resolution). The intrinsic parameters obtained through Zhang’s method [12] are shown in Table 2 and the extrinsic parameters are shown in Table 3. The calibration of each camera achieved an accuracy of 0.1 pixel based on 21 images (1–11 are obtained on the turntable and 12–21 prepared beforehand; see Figure 9). The rotation matrix calibration achieved an accuracy of <0.1° for each Euler angle, and the position vector calibration achieved an accuracy of <0.6 mm for each position vector component.

## 5. Navigation Experiment

**Figure 10:**
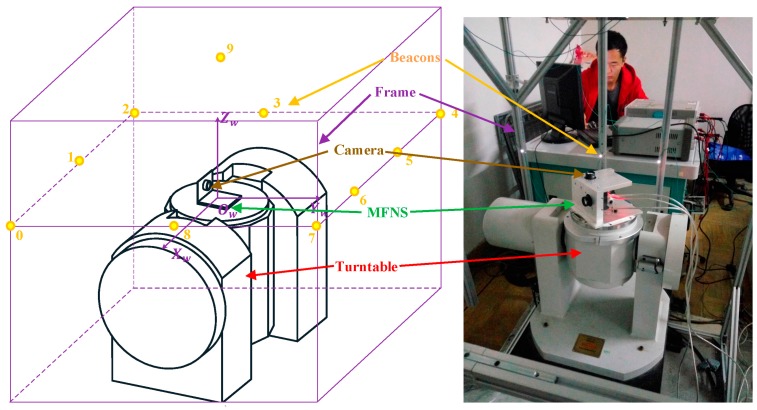
Architecture of the navigation experiment.

A complete model for the navigation system is established after calibration. Then, a demonstration experiment is conducted to confirm the validity of the calibration method and evaluate the accuracy of the system. The architecture of the experiment is shown in Figure 10.

Ten LED beacons are fixed on the steel frame with an apparent size of approximately 1 × 1 × 1.5 m^3^ around the turntable. As the position of the beacons in the world coordinate are known, the position and attitude of the MFNS can be calculated using the imaging model (Equation (3)), while plenty beacons are observed by the MFNS. The experiment is designed as follows to verify the accuracy of the navigation result, namely, the calculated pose (three-axis position and three-axis attitude) of the MFNS. First, the origin of the world coordinate is defined as the center of the turntable platform, which is the origin of the MFNS body coordinate in the initial pose, and the three axes of the world coordinates *X_t_*, *Y_t_*, and *Z_t_* are defined parallel to the axes of the world coordinates *X_w_*, *Y_w_*, and *Z_w_*, respectively. Then, the position of the beacons is measured, as shown in Table 4. Second, the turntable is controlled to rotate around the *Z_t_* axis, while the MFNS moves with the turntable, takes pictures of the surrounding environment, and calculates the pose of the MFNS based on the beacons observed.

**Figure 11:**
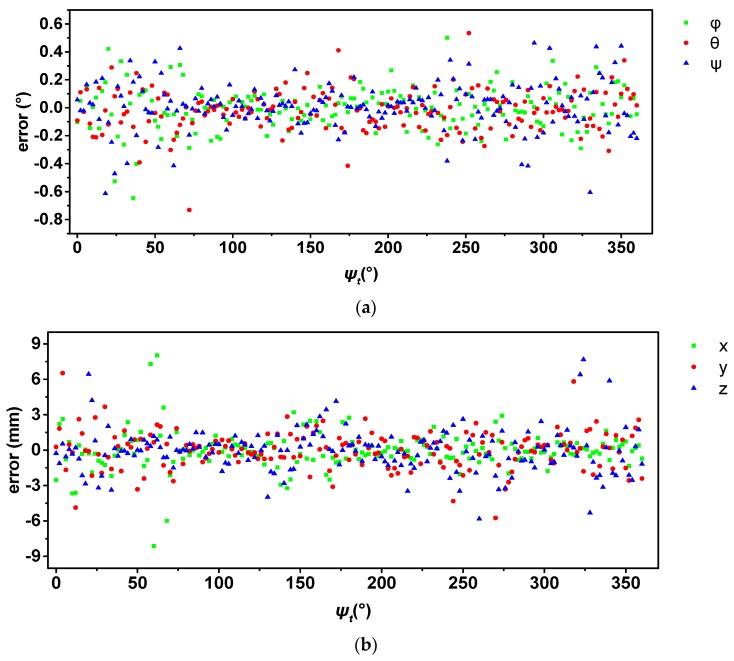
Results of the navigation experiment: (**a**) attitude error and (**b**) position error.

Finally, based on the definition of the coordinates, the position of the turntable platform center does not move during the rotation. The true value of the MFNS position is (0, 0, 0) and the true attitude of the MFNS is (0, 0, *ψ_t_*), where *ψ_t_* is the angle given by the turntable around its *Z_t_* axis. Therefore, the pose error is obtained as the difference between the calculated pose and the true value. Figure 11 and Table 5 show the navigation results.

The analysis of experiment result, discussion on existing problems and possible reasons, ways to improve, and future work are as follows:

1. As the origin of the MFNS coordinate, *O_b_* is defined on the mounting surface of the turntable, and the position of the MFNS is constant (0, 0, 0) while the turntable rotates around its *Z_t_* axis. Similarly, *φ* and *θ* remain 0 and *ψ* varies linearly while the turntable rotates uniformly under the ideal condition. The experiment results show that the MFNS worked properly during validation; the stand deviations of the three-axis position error are 1.60, 1.61, and 1.83 mm, respectively; and the stand deviations of the three-axis attitude error are 0.15°, 0.15°, and 0.17°, respectively.
2. The calibration method proposed for multiple camera systems deals with calibration results from the single-camera calibration method. In Section 3, we mainly utilize the LSQ for formula derivation and finding the optimization solution. This approach makes understanding the main idea and the process of our method easier, and the experiment results show that the performance of the MFNS after calibration is acceptable. However, the LSQ may not be the most accurate method to solve the problem, because any error will propagate during the process. For instance, when calculating $C_{x}^{b}$ and $C_{y}^{b}$, according to Equations (A11) and (A12), the results are based on $C_{t}^{w}$, which is the optimal solution of Equation (A10). More in-depth work may focus on better ways to obtain optimal solutions on system extrinsic parameters.
3. Given that the imaging model of the MFNS was built in Section 2, research on the navigation algorithm of the MFNS should be carried out afterwards. The navigation experiment is only conducted to verify the accuracy of the system calibration result, because we simply use the Newton iteration method to find a numerical solution of navigation parameters. The problem of beacon pattern recognition and multiple solutions are ignored by manually matching the beacons and choosing the iteration initial value. Furthermore, a solution in a pose that is less than three beacons observed by the MFNS is difficult to obtain. Therefore, a complete navigation algorithm system is our goal for future work, including solution strategy, error propagation analysis, beacon distribution, and optimized methods.

## 6. Conclusions

The basics of the MFNS, including its system design, mathematical model, and error analysis, is proposed in detail in this paper. This approach will be a reference to further develop and study other multiple-camera vision systems. A novel calibration method based on error analysis, namely, CPC for MFNS, is proposed. A calibration experiment is conducted where intrinsic and extrinsic parameters are simultaneously obtained utilizing the method proposed. The navigation experiment shows that ideal calibration results are achieved. This method can be integrated into a toolkit and used for other vision systems, especially multiple-camera systems or FOV-separated systems.

## Figures and Tables

**Table 1:** Parameters for MC simulation on MFNS error analysis.

**Camera Parameter**					
*α_x_* (pixel)	*α_y_* (pixel)	*u*_0_ (pixel)	*v*_0_ (pixel)		
1600	1600	640	512		
**Extrinsic Parameter of MFNS**					
*φ* (°)	*θ* (°)	*ψ* (°)	*T_x_* (mm)	*T_y_* (mm)	*T_z_* (mm)
0	0	0	0	0	0
**Beacon Position**					
*X_w_* (mm)	*Y_w_* (mm)	*Z_w_* (mm)			
0–1000	-	0–1000			
**Number of MC Simulation Trials for Each Group of Parameters**					
10,000					

**Table 2:** Calibration result of intrinsic parameters of camera X, Y and Z.

Parameter	Camera X		Camera Y		Camera Z	
	Calibration Result	Error	Calibration Result	Error	Calibration Result	Error
*α_x_*	1599.26136	1.78966	1605.35286	1.66249	1611.21596	2.14183
*α_y_*	1599.93302	1.64101	1603.54359	1.73608	1610.79607	2.14219
*u*_0_	632.61591	1.62023	619.71227	1.80177	649.51210	1.39920
*v*_0_	522.17870	1.61823	505.99361	1.93821	531.29472	1.34749
*α*	0.00000	0.00000	0.00000	0.00000	0.00000	0.00000
*kc*_1_	−0.13013	0.00336	−0.11276	0.00301	−0.10481	0.00338
*kc*_2_	0.28701	0.02243	0.01776	0.01776	0.15881	0.02341
*kc*_3_	−0.00040	0.00029	−0.00031	0.00034	−0.00137	0.00021
*kc*_4_	−0.00004	0.00031	0.00057	0.00030	0.00184	0.00022
*kc*_5_	0.00000	0.00000	0.00000	0.00000	0.00000	0.00000
Pixel error	(0.11711, 0.10363)		(0.11597, 0.12652)		(0.11593, 0.11232)	

**Table 3:** Calibration result of the extrinsic parameters.

Rotation Matrix				Position Vector			
Matrix	Euler Angle	Calibration Result (°)	Error (°)	Vector	Component	Calibration Result (mm)	Error (mm)
$C_{t}^{w}$	*α*	−0.0110	0.0485	$r_{wb}^{w}$	$r_{wb,x}^{w}$	33.8407	0.2932
	*β*	0.1263	0.0657		$r_{wb,y}^{w}$	−128.9401	0.2932
	*γ*	0.0861	0.0443		$r_{wb,z}^{w}$	576.9100 (measured)	0.2456
$C_{x}^{b}$	*φ_x_*	−0.5788	0.0413	$r_{wt}^{w}$	$r_{wt,x}^{w}$	34.0949	0.1496
	*θ_x_*	−90.3523	0.0990		$r_{wt,y}^{w}$	−128.9281	0.1496
	*ψ_x_*	0.0395	0.0153		$r_{wt,z}^{w}$	−684.7134	0.1496
$C_{y}^{b}$	*φ_y_*	90.1636	0.0301	$r_{bx}^{b}$	$r_{bx,x}^{b}$	76.1319	0.2529
	*θ_y_*	−0.5501	0.0920		$r_{bx,y}^{b}$	−36.8373	0.4498
	*ψ_y_*	0.1925	0.0157		$r_{bx,z}^{b}$	78.8949	0.4959
$C_{z}^{b}$	*φ_z_*	−0.1076	0.0485	$r_{by}^{b}$	$r_{by,x}^{b}$	−32.5004	0.5358
	*θ_z_*	−0.7211	0.0657		$r_{by,y}^{b}$	75.7149	0.0825
	*ψ_z_*	1.1690	0.0443		$r_{by,z}^{b}$	76.9967	0.1963
				$r_{bz}^{b}$	$r_{bz,x}^{b}$	34.1604	0.3097
					$r_{bz,y}^{b}$	30.8251	0.3097
					$r_{bz,z}^{b}$	119.7096	0.2932

**Table 4:** Position of beacons.

Number of Beacons	Position of Beacons (mm)		
	*x*	*y*	*z*
0	487.4734	−720.0265	240.8359
1	−51.2618	−737.6155	164.7393
2	−366.1525	−711.8317	131.2681
3	−458.8302	−215.2964	−69.2627
4	−454.6380	410.3959	−60.6549
5	−117.4392	296.0537	83.0754
6	182.4478	279.1964	143.2510
7	481.8346	252.9609	217.5775
8	647.3834	−127.6167	178.7407
9	−122.5798	−44.9064	562.5394
Estimated position accuracy:		0.3963 mm	

**Table 5:** Results of the navigation experiment.

Parameter	Mean Error	Standard Deviation
Roll (*φ*/°)	−0.0030	0.1541
Pitch (*θ*/°)	−0.0192	0.1497
Yaw (*ψ*/°)	0.0066	0.1729
*x* position (mm)	−0.0553	1.6043
*y* position (mm)	0.0275	1.6108
*z* position (mm)	−0.0669	1.8292

**Figure A1:**
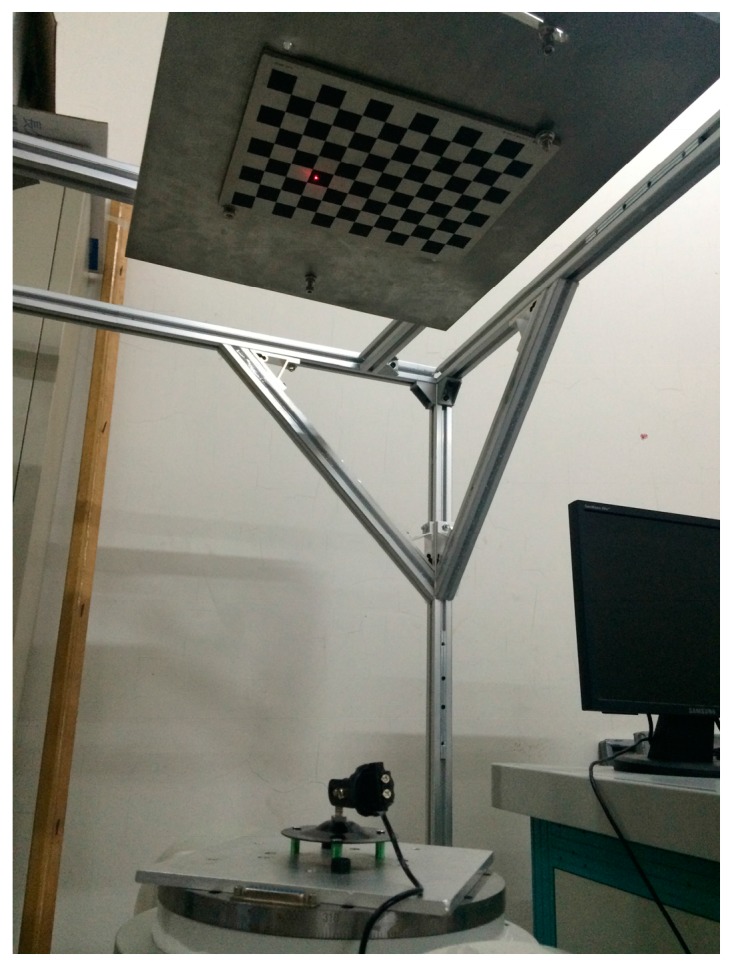
Laser is installed on the turntable to adjust the orientation of the checkerboard.

## Appendix A. CPC Method for MFNS Calibration

### Appendix A.1. Calibration of Rotation Matrix $C_{k}^{b}$

The proposed calibration process needs the aid of a two-axis turntable, as shown in Figure 7. According to the definition of the coordinates, the rotation matrix between the two coordinates $C_{t}^{b}$ is an identity matrix when the MFNS is mounted to the turntable.

The checkerboard is fixed on the frame (Figure 7). When the system is in the initial pose, the checkerboard is in the FOV of camera Z, and the transformation between the coordinate systems at this moment is as follows:

(A1) $$C_{z}^{w}=C_{t}^{w}\cdot C_{b}^{t}\cdot C_{z}^{b}=C_{t}^{w}\cdot C_{z}^{b}$$

$C_{z}^{w}$ is obtained using Zhang’s calibration method based on the imaging model. $C_{z}^{b}$ is the rotation matrix to be calibrated. $C_{t}^{w}$ is unknown and coupled with $C_{z}^{b}$, making the value of the two matrices difficult to calculate. To solve this problem, an IMU and a miniature solid-state laser are utilized to align the turntable coordinate and the world coordinate.

The two-axis turntable needs a strict level adjustment before use because of the task demand of the inertial device calibration. Therefore, the *O_w_X_w_Y_w_* plane is nearly parallel to the *O_t_X_t_Y_t_* plane after level adjustment of the checkerboard with the aid of the IMU’s gravity measurement. The heading of the world coordinate can be adjusted afterwards by fixing the laser on the turntable platform. The alignment of the trace of the laser spot on the checkerboard can easily be done when the laser rotates with the platform around the *X_t_* axis of the turntable coordinate. This alignment points to the direction of the *Y_t_* axis (the details of the coordinate alignment can be seen in Appendix A.3). According to [35], when the Euler angles represent small quantities where small angle approximation is valid, the rotation matrix can be simplified. For instance, the difference between an angle less than 1.5° in radian and its sine is less than 3 × 10^−6^. After the alignment, the Euler angles corresponding to $C_{t}^{w}$ are small quantities less than 0.5°, and the rotation matrix can be simplified as:

(A2) $$C_{t}^{w}=\left[\begin{array}{ccc} 1 & \gamma & -\beta \\ -\gamma & 1 & \alpha \\ \beta & -\alpha & 1 \end{array}\right]$$

where *α*, *β,* and *γ* are the Euler angles corresponding to $C_{t}^{w}$. Each of these angles is in small quantities.

Note that the Euler angles corresponding to $C_{z}^{b}$ are also in small quantities (ensured by the finishing machining of the MFNS structure frame), namely, *φ*, *θ,* and *ψ*. $C_{z}^{b}$ is written as:

(A3) $$C_{z}^{b}=\left[\begin{array}{ccc} 1 & \psi & -\theta \\ -\psi & 1 & \varphi \\ \theta & -\varphi & 1 \end{array}\right]$$

Let the turntable platform rotate an angle *Ω* around the *Z_t_* axis so that camera Z of the MFNS rotates to a position *Z**′* and the checkerboard is still in the FOV of camera Z. The transformation of the coordinates is as follows:

(A4) $$C_{z\prime }^{w}=C_{t}^{w}\cdot C_{b}^{t}\cdot C_{b\prime }^{b}\cdot C_{z\prime }^{b\prime }$$

where:

(A5) $$C_{b\prime }^{b}=\left[\begin{array}{ccc} \cos\Omega & -\sin\Omega & 0\\ \sin\Omega & \cos\Omega & 0\\ 0 & 0 & 1 \end{array}\right]$$

Noting that $C_{b}^{t}=I_{3}$ and $C_{z}^{b}=C_{z\prime }^{b\prime }$, we get the following:

(A6) $$C_{z\prime }^{w}=C_{t}^{w}\cdot C_{b\prime }^{b}\cdot C_{z}^{b}$$

On the basis of Equations (A2), (A3), (A5) and (A6), we get:

(A7) $$C_{z\prime }^{w}=\left[\begin{array}{ccc} 1 & \gamma & -\beta \\ -\gamma & 1 & \alpha \\ \beta & -\alpha & 1 \end{array}\right]\left[\begin{array}{ccc} \cos\Omega & -\sin\Omega & 0\\ \sin\Omega & \cos\Omega & 0\\ 0 & 0 & 1 \end{array}\right]\left[\begin{array}{ccc} 1 & \psi & -\theta \\ -\psi & 1 & \varphi \\ \theta & -\varphi & 1 \end{array}\right]$$

With the higher-order terms neglected, Equation (A7) can be written as:

(A8) $$C_{z\prime }^{w}=\left[\begin{array}{ccc} \cos\Omega +\gamma \cdot \sin\Omega +\psi \cdot \sin\Omega & \psi \cdot \cos\Omega +\gamma \cdot \cos\Omega -\sin\Omega & -\theta \cdot \cos\Omega -\varphi \cdot \sin\Omega -\beta \\ -\gamma \cdot \cos\Omega +\sin\Omega -\psi \cdot \cos\Omega & \psi \cdot \sin\Omega +\cos\Omega +\gamma \cdot \sin\Omega & -\theta \cdot \sin\Omega +\varphi \cdot \cos\Omega +\alpha \\ -\alpha \cdot \sin\Omega +\beta \cdot \cos\Omega +\theta & -\alpha \cdot \cos\Omega -\beta \cdot \sin\Omega -\varphi & 1 \end{array}\right]$$

Similarly, when the turntable platform rotates an angle *ω* around the *X_t_* axis so that camera Z of the MFNS rotates to a position *Z″* and the checkerboard is still in the FOV of camera Z, the transformations of the coordinates are:

(A9) $$C_{z\prime \prime }^{w}=\left[\begin{array}{ccc} 1 & \psi +\gamma \cdot \cos\omega -\beta \cdot \sin\omega & -\theta -\gamma \cdot \sin\omega -\beta \cdot \cos\omega \\ -\gamma -\theta \cdot \sin\omega -\psi \cdot \cos\omega & \cos\omega +\alpha \cdot \sin\omega +\varphi \cdot \sin\omega & \varphi \cdot \cos\omega -\sin\omega +\alpha \cdot \cos\omega \\ \beta -\psi \cdot \sin\omega +\theta \cdot \cos\omega & -\alpha \cdot \cos\Omega +\sin\omega -\varphi \cdot \cos\Omega & \varphi \cdot \sin\omega +\alpha \cdot \sin\omega +\cos\omega \end{array}\right]$$

On the basis of Equations (A8) and (A9), *φ*, *θ,* and *ψ,* as well as *α*, *β,* and *γ* can be calculated using the least-squares method:

(A10) $$\left[\begin{array}{cccccc} -\sin\Omega & \cos\Omega & 0 & 0 & 1 & 0\\ -\cos\Omega & -\sin\Omega & 0 & -1 & 0 & 0\\ 0 & -1 & 0 & -\sin\Omega & -\cos\Omega & 0\\ 1 & 0 & 0 & \cos\Omega & -\sin\Omega & 0\\ \ldots \\ 0 & -\sin\omega & \cos\omega & 0 & 0 & 1\\ 0 & -\cos\omega & -\sin\omega & 0 & -1 & 0\\ 0 & 0 & -1 & 0 & -\sin\omega & -\cos\omega \\ 0 & 1 & 0 & 0 & \cos\omega & -\sin\omega \\ \ldots \end{array}\right]\left[\begin{array}{c} \alpha \\ \beta \\ \gamma \\ \varphi \\ \theta \\ \psi \end{array}\right]=\left[\begin{array}{c} C_{z\prime 31}^{w(1)}\\ C_{z\prime 32}^{w(1)}\\ C_{z\prime 13}^{w(1)}\\ C_{z\prime 23}^{w(1)}\\ \ldots \\ C_{z\prime \prime 12}^{w(1)}\\ C_{z\prime \prime 13}^{w(1)}\\ C_{z\prime \prime 21}^{w(1)}\\ C_{z\prime \prime 31}^{w(1)}\\ \ldots \end{array}\right]$$

Let the turntable rotate so that the checkerboard is in the FOV of cameras X and Y. The transformations of the coordinates are:

(A11) $$C_{x\prime }^{w}=C_{t}^{w}\cdot C_{b\prime }^{b}\cdot C_{x\prime }^{b\prime }$$

(A12) $$C_{y\prime }^{w}=C_{t}^{w}\cdot C_{b\prime \prime }^{b}\cdot C_{y\prime }^{b\prime \prime }$$

where $C_{x\prime }^{w}$ and $C_{y\prime }^{w}$ are obtained by Zhang’s method, $C_{t}^{w}$ is already known through Equation (A10), and $C_{b\prime }^{b}$ and $C_{b\prime \prime }^{b}$ are manually controlled. The optimal solutions of $C_{x}^{b}$ and $C_{y}^{b}$ can be obtained as $C_{x}^{b}=C_{x\prime }^{b\prime }$ and $C_{y}^{b}=C_{y\prime }^{b\prime \prime }$.

Thus far, the rotation matrices are all obtained.

### Appendix A.2. Calibration of Vector **r**_i_

Vectors in this paper are appointed as follows: $r_{ij}^{k}$ represents the vector from coordinate *i*’s origin *O_i_* to coordinate *j*’s origin *O_j_* and expressed in coordinate *k*. When the checkerboard is in the FOV of camera Z, the position relationship among the coordinate origins is:

(A13) $$r_{wz}^{w}=r_{wb}^{w}+r_{bz}^{w}=r_{wt}^{w}+r_{tb}^{w}+r_{bz}^{w}$$

If the turntable rotates only around the *Z_t_* axis, then the position of *O_b_* does not change. Thus:

(A14) $$r_{wz\prime }^{w}=r_{wb}^{w}+r_{b\prime z\prime }^{w}=r_{wb}^{w}+C_{b\prime }^{w}\cdot r_{b\prime z\prime }^{b\prime }=r_{wb}^{w}+C_{b\prime }^{w}\cdot r_{bz}^{b}$$

where *z′* and *b′* represent the new positions of camera Z and the MFNS respectively.

Otherwise, the turntable rotates freely. Given that the position of *O_t_* does not change, we get:

(A15) $$r_{wz\prime \prime }^{w}=r_{wt}^{w}+r_{tb\prime \prime }^{w}+r_{b\prime \prime z\prime \prime }^{w}=r_{wt}^{w}+C_{b\prime \prime }^{w}\cdot r_{tb\prime \prime }^{b\prime \prime }+C_{b\prime \prime }^{w}\cdot r_{bz}^{b}$$

On the basis of Equation (A14), through multiple images when the turntable rotates only around the *Z_t_* axis, we get the following:

(A16) $$r_{wz\prime_{\left(i\right)}}^{w}-r_{wz\prime_{\left(j\right)}}^{w}=(C_{b\prime_{\left(i\right)}}^{w}-C_{b\prime_{\left(j\right)}}^{w})\cdot r_{bz}^{b}$$

Let $r_{bz}^{b}={\left[z_{x}\;z_{y}\;z_{z}\right]}^{T}$, *z_x_* and *z_y_* can be obtained by the least squares method (LSQ) based on Equation (A16).

Let $r_{wb}^{w}={\left[b_{x}\;b_{y}\;b_{z}\right]}^{T}$. As *b_z_* and *z_z_* are coupled and cannot be calculated by rotating the turntable, *b_z_* needs to be measured with a vernier caliper. Then *b_x_*, *b_y_*, and *z_z_* are obtained by LSQ based on Equation (A14).

Afterwards, $r_{wt}^{w}$ and $r_{tb}^{b}$ are calculated to prepare for the calibration of vectors $r_{bx}^{b}$ and $r_{by}^{b}$ based on Equation (A15).

The turntable is then controlled so that cameras X and Y can take photographs of the checkerboard. The position vectors are then given by:

(A17) $$r_{wx\prime }^{w}=r_{wt}^{w}+C_{b\prime }^{w}\cdot r_{tb}^{b}+C_{b\prime }^{w}\cdot r_{bx}^{b}$$

(A18) $$r_{wy\prime }^{w}=r_{wt}^{w}+C_{b\prime }^{w}\cdot r_{tb}^{b}+C_{b\prime }^{w}\cdot r_{by}^{b}$$

With all the other parameters known, the optimal solutions for $r_{bx}^{b}$ and $r_{by}^{b}$ can be obtained.

### Appendix A.3. Operational Method for Coordinates’ Alignment

Two coordinates, *O_w_X_w_Y_w_Z_w_* and *O_t_X_t_Y_t_Z_t_*, need to be aligned before the system is calibrated. In this study, an operational method composed of two steps is introduced.

#### **Step 1:** **Level adjustment of the checkerboard**

##### Device required

A high-accuracy accelerometer.

##### Principle

The biaxial turntable used in the experiment has a mounting surface, whose level is ensured through rigorous adjustment because of the need to calibrate the inertial device. Thus, the level adjustment of the checkerboard is accomplished by making use of gravity. Specifically, place the accelerometer on the mounting surface of the turntable and the back of checkerboard to measure the specific force, and adjust the checkerboard so that the specific force measurements *f_tz_* and *f_wz_* are:

$$f_{tz}=f_{wz}$$

As a result, the *O_w_X_w_Y_w_* plane is parallel to the *O_t_X_t_Y_t_* plane, and there is only a difference of yaw angle *ψ_tw_* between the two coordinates.

#### **Step 2:** **Orientation adjustment of the world coordinate**

##### Device required

A point light laser.

##### Principle

The laser is installed as shown in Figure A1. The reference frame is set as turntable coordinate *O_t_X_t_Y_t_Z_t_*. Assume the laser beam incident point is *P*_0_ (*a*, *b*, *c*) and the beam is a straight line with a direction vector ***n*** = (*m*, *n*, 1)^T^. The equation of laser beam *l*_0_ in the turntable coordinate is written as:

$$\frac{x-a}{m}=\frac{y-b}{n}=z-c$$

When the laser rotates an angle of *θ* with the turntable around its *Y_t_*, its direction vector n′ is given by:

$$n\prime =\left[\begin{array}{ccc} 1 & 0 & 0\\ 0 & \cos\varphi & \sin\varphi \\ 0 & -\sin\varphi & \cos\varphi \end{array}\right]\cdot \left[\begin{array}{c} m\\ n\\ 1 \end{array}\right]=\left[\begin{array}{c} m\\ n\cos\varphi +\sin\varphi \\ -n\sin\varphi +\cos\varphi \end{array}\right]$$

Similarly, $\left[\begin{array}{ccc} 1 & 0 & 0\\ 0 & \cos\varphi & \sin\varphi \\ 0 & -\sin\varphi & \cos\varphi \end{array}\right]\cdot \left[\begin{array}{c} a\\ b\\ c \end{array}\right]=\left[\begin{array}{c} a\\ b\cos\varphi +c\sin\varphi \\ -b\sin\varphi +c\cos\varphi \end{array}\right]$; thus, the coordinate of incident point *P* is (a,bcosφ+csinφ,−bsinφ+ccosφ). Thus, the equation of laser beam *l* is as follows:

$$\frac{x-a}{m}=\frac{y-(b\cos\varphi +c\sin\varphi )}{n\cos\varphi +\sin\varphi }=\frac{z-(-b\sin\varphi +c\cos\varphi )}{-n\sin\varphi +\cos\varphi }$$

Assume that the vertical distance between the checkerboard and the turntable mounting surface is *h*, and the intersection point of the laser beam and the checkerboard *P*_1_ is:

$$\left(m\frac{h-(-b\sin\varphi +c\cos\varphi )}{-n\sin\varphi +\cos\varphi }+a,(n\cos\varphi +\sin\varphi )\frac{h-(-b\sin\varphi +c\cos\varphi )}{-n\sin\varphi +\cos\varphi }+(b\cos\varphi +c\sin\varphi ),h\right)$$

From the coordinate of *P*_1_, when *m* = 0, the trace of *P*_1_ on the checkerboard will be a straight line, which is parallel to the *Y_t_* axis of the turntable. Therefore, we can adjust the direction of the laser and the checkerboard simultaneously while rotating the turntable around its *X_t_* axis, so that the trace of the laser dot aligns with the grid line of the checkerboard. As a result, the *Y_w_* and *Y_t_* axes are parallel to each other, and the alignment of the two coordinates is complete.
